# KOH-Activated Hierarchical Porous Carbon Derived from *Suaeda salsa* for Supercapacitor Electrodes: Pore-Structure Evolution and Electrochemical Behavior

**DOI:** 10.3390/nano16211412

**Published:** 2026-11-03

**Authors:** Yihan Zhao, Puyu Chen, Antong Ren, Zhuofan Wu, Hao Zhou, Xu Zhao, Xiaojing Yang

**Affiliations:** School of Chemical Engineering, Ocean and Life Sciences, Dalian University of Technology, Dalian 116024, China; 15931913390@mail.dlut.edu.cn (Y.Z.); intelligent@mail.dlut.edu.cn (P.C.); 2024202030058@whu.edu.cn (A.R.); wuzhuofan1212@163.com (Z.W.); zhouhao@dlut.edu.cn (H.Z.); zhaoxv@dlut.edu.cn (X.Z.)

**Keywords:** *Suaeda salsa*, biomass-derived porous carbon, KOH activation, supercapacitor, electrochemical double-layer capacitor

## Abstract

The increasing demand for sustainable electrochemical energy-storage materials has stimulated considerable interest in biomass-derived porous carbons. In this study, hierarchical porous carbon was prepared from *Suaeda salsa*, a renewable halophytic biomass, through thermal pretreatment, carbonization, and KOH activation. The effects of thermal treatment and KOH activation on the pore structure, morphology, surface characteristics, and electrochemical behavior of the resulting carbons were systematically investigated. The results demonstrate that moderate KOH activation substantially opens the carbon framework and promotes the development of accessible porosity while retaining a finite meso/macro-pore network that can facilitate electrolyte transport. Among the investigated conditions, the intermediate activation level provided the most favorable balance between micropore generation, transport accessibility, and framework preservation, leading to the best electrochemical response. The combined structural and electrochemical analyses establish a clear processing–pore structure–property relationship and indicate that electrochemical performance cannot be optimized simply by increasing activation severity. Instead, rational control of hierarchical porosity is important for achieving effective charge storage and ion transport in biomass-derived carbon electrodes. The study provides a sustainable route for converting coastal halophytic biomass into functional porous carbon materials for electrochemical energy-storage applications.

## 1. Introduction

The growing integration of intermittent renewable energy sources has increased the demand for electrochemical energy-storage technologies that can deliver high power and withstand rapid charge–discharge cycling [1,2,3,4,5,6]. Electric double-layer capacitors (EDLCs) are attractive in this context because of their high power density, fast response, and long cycle life [4,5,6,7]. Carbon materials, including activated carbon, carbon fibers, aerogels, graphite, graphene, and carbon nanotubes, are widely used as EDLC electrodes because their accessible surface area and pore architecture can support reversible ion adsorption [8,9,10,11,12,13,14,15,16].

For porous-carbon EDLC electrodes, a high specific surface area alone does not guarantee high capacitance. Micropores provide most of the ion-storage surface, whereas mesopores and larger channels can shorten diffusion paths and act as ion-buffering/transport domains [17,18,19,20]. Importantly, the electrochemically accessible surface depends on the relationship between pore width, electrolyte-ion size, and solvation state. Seminal studies on carbide-derived and activated carbons showed that capacitance can increase when pore sizes approach the dimensions of the electrolyte ions, demonstrating that pore accessibility and confinement effects are as important as the nominal BET area [19,21,22]. Accordingly, hierarchical porous carbons that combine abundant small pores for charge storage with a finite population of larger transport pores are often preferable to materials designed solely to maximize total surface area [20,23].

Biomass-derived carbons offer a sustainable route to porous electrode materials because renewable precursors can provide intrinsic microstructures and heteroatom-containing surface functionalities while reducing dependence on fossil-derived carbon sources [23,24,25]. However, the electrochemical properties of biomass-derived carbons are highly sensitive to precursor composition, carbonization temperature, and activation severity. A systematic evaluation of these processing variables is therefore necessary to distinguish the contribution of the biomass morphology from that of chemical activation.

*Suaeda salsa* is a halophytic plant widely distributed in saline-alkali environments, including coastal Panjin. Its lignocellulosic framework and naturally developed internal channels make it a potentially useful carbon precursor [20,26,27]. Compared with conventional agricultural residues, the use of a salt-tolerant coastal biomass broadens the range of renewable feedstocks available for porous-carbon preparation.

In this work, *Suaeda salsa* was thermally converted and KOH-activated to obtain porous carbon electrodes [28]. The temperature dependence of the unactivated carbon was first examined at 800, 900, and 1000 °C, after which the effect of the KOH-to-pretreated-powder mass ratio (0.2, 0.5, and 1.0) was evaluated at 800 °C. This study focuses on elucidating the processing–pore structure–property relationship and identifying an activation condition that balances pore development, ion-accessible transport pathways, and capacitive response.

## 2. Materials and Methods

### 2.1. Materials

*Suaeda salsa* (5–10 cm in length) used in this study was harvested from coastal mudflats in the Panjin City, China. Per batch, approximately 500 g of raw *Suaeda salsa* was used, and after cutting, approximately 100 g of usable stem segments (5–10 cm in length) were obtained. Potassium hydroxide (KOH) and acetylene black were purchased from Tianjin Damao Chemical Reagent Factory in Tianjin CIty, China. Ethanol was purchased from Panjin Tianyuan Pharmaceutical Co., Ltd. in Panjin CIty, China. Concentrated hydrochloric acid was purchased from Tianjin Yuanli Chemical in Tianjin CIty, China. Nafion (5 wt.%) was purchased from Sigma-Aldrich Corporation, St. Louis, MO, USA. All chemicals were of analytical grade.

### 2.2. Preparation of Suaeda salsa-Derived Porous Carbon

The collected *Suaeda salsa* biomass was thoroughly washed and then dried in a vacuum oven at 60 °C for 8 h to remove moisture. We then placed it in a muffle furnace and thermally pretreated it in air by heating it to 300 °C at a rate of 3 °C min^−1^ and maintaining this temperature for 2 h. After cooling to room temperature, the treated material was ground for 20 min to obtain a homogeneous powder.

Temperature-screening series: A portion of the thermally pretreated powder, without added activator, was heated under flowing N_2_ (approximately 100 mL min^−1^) at 5 °C min^−1^ to 800, 900, or 1000 °C and held for 2 h at the target temperature.

KOH-activation series: The thermally pretreated powder was mixed with KOH at KOH-to-pretreated-powder mass ratios of 0.2, 0.5, and 1.0. Each mixture was dispersed in 20 mL of deionized water, stirred thoroughly, and held at approximately 20 °C for 12 h. The impregnated mixtures were dried in a vacuum oven at 60 °C for 10 h and then heated under flowing N_2_ (approximately 100 mL min^−1^) at 800 °C (5 °C min^−1^) for 2 h. The resulting samples are denoted JPC-800-X, where X is the KOH-to-pretreated-powder mass ratio.

### 2.3. Purification

The as-prepared JPC-T-X samples were treated with 2 mol L^−1^ HCl (10 mL of 2 mol L^−1^ HCl per gram of solid) for 12 h to remove residual inorganic species.

After acid treatment, the solids were separated by centrifugation and repeatedly washed with deionized water and ethanol until the supernatant was approximately neutral (pH ≈ 7). The purified materials were dried in a vacuum oven at 80 °C for 10 h and sealed for storage.

### 2.4. Material Characterization

The morphology of the carbon materials was examined by field-emission scanning electron microscopy (Nova 450 NanoSEM, FEI Company, Hillsboro, OR, USA) and transmission electron microscopy (FEI Tecnai G2 F30 STWIN, FEI Company, Hillsboro, OR, USA). X-ray diffraction (XRD) patterns were collected on a Shimadzu XRD-7000 (Shimadzu, Kyoto, Japan) diffractometer over 10–90°. N_2_ adsorption–desorption isotherms were measured at 77 K using an Autosorb iQ C analyzer (Quantachrome Instruments, Boynton Beach, FL, USA). The BET method was used to determine the specific surface area. Pore-volume and pore-size parameters are reported from the N_2_ adsorption–desorption analysis used in this study. Surface composition and chemical states were analyzed by X-ray photoelectron spectroscopy (XPS; ESCALAB 250Xi, Thermo Fisher Scientific, East Grinstead, UK) using an Al Kα source. Raman spectra were collected with a Renishaw inVia spectrometer to evaluate the disorder/graphitic characteristics of the carbons.

### 2.5. Electrode Preparation

For three-electrode measurements, *Suaeda salsa*-derived carbon and acetylene black were mixed in a mass ratio of 8.5:1.5 (10 mg total solids). An ink was prepared by adding 0.5 mL of anhydrous ethanol, 0.5 mL of deionized water, and 20 μL of 5 wt.% Nafion solution, followed by ultrasonication for 2 h at room temperature. A 50 μL aliquot was coated onto carbon paper (1 cm × 1 cm; substrate mass: 7.7–8.3 mg) and dried at 30 °C for 2 h. The nominal active-carbon loading used for gravimetric normalization was 0.2–0.5 mg per working electrode, excluding the carbon-paper substrate.

For the symmetric two-electrode configuration, the carbon/acetylene-black ink was prepared using the same 8.5:1.5 mass ratio and solvent/binder composition. A 57 μL aliquot was coated onto the 12 mm carbon-paper electrode (substrate mass: 8.7–9.3 mg) and dried at 30 °C for 2 h. The nominal active-carbon loading used for normalization was approximately 0.2 mg per electrode, excluding the carbon-paper substrate.

### 2.6. Electrochemical Characterization

Electrochemical measurements were performed using a CHI660E workstation (Shanghai Chenhua Instrument Co., Ltd., Shanghai, China) in a three-electrode configuration with JPC-coated carbon paper as the working electrode, a Pt wire as the counter electrode, and Hg/HgO as the reference electrode in 6 mol L^−1^ KOH. For comparison with the literature, the working-electrode potentials in the three-electrode configuration. are reported on the RHE scale after conversion from Hg/HgO using the convention adopted in this study: E_RHE = E_Hg/HgO + 0.965 V at 25 °C (equivalently, E_Hg/HgO = E_RHE − 0.965 V). The illustration of the two-electrode system retains the original Hg/HgO potential scale, as indicated in the figure caption. CV measurements were conducted at scan rates of 5–160 mV s^−1^, and GCD measurements were conducted at current densities of 0.5–10 A g^−1^. Long-term cycling of JPC-800-0.5 was performed at 5 A g^−1^. The specific capacitance was calculated from the discharge branch using Equation (1).

C_s_ = IΔt/(mΔV) (1)

For the three-electrode configuration, the specific capacitance was calculated from the discharge branch using Equation (1), where I is the discharge current (A), Δt is the discharge time (s), ΔV is the effective discharge voltage window after excluding the initial IR drop (V), and m is the mass of active carbon used for normalization (g).

For the symmetric two-electrode device, the device-specific capacitance was calculated as

C = 2 IΔt/(mΔV) (F g^−1^) (2)

where m is the active-carbon mass on the single electrode. The energy density (E, Wh kg^−1^) and power density (P, W kg^−1^) were calculated using Equations (3) and (4), respectively. Here, ΔV is the full-cell discharge voltage window after excluding the initial IR drop, and Δt is the discharge time. These device-level definitions are distinct from the three-electrode equation above.

E = CΔV^2^/(8 × 3.6) (Wh kg^−1^) (3)

P = 3600 E/Δt (W kg^−1^) (4)

## 3. Results and Discussion

### 3.1. Effect of Pyrolysis Temperature on the Pore Structure and Capacitive Performance

**Figure 1:**
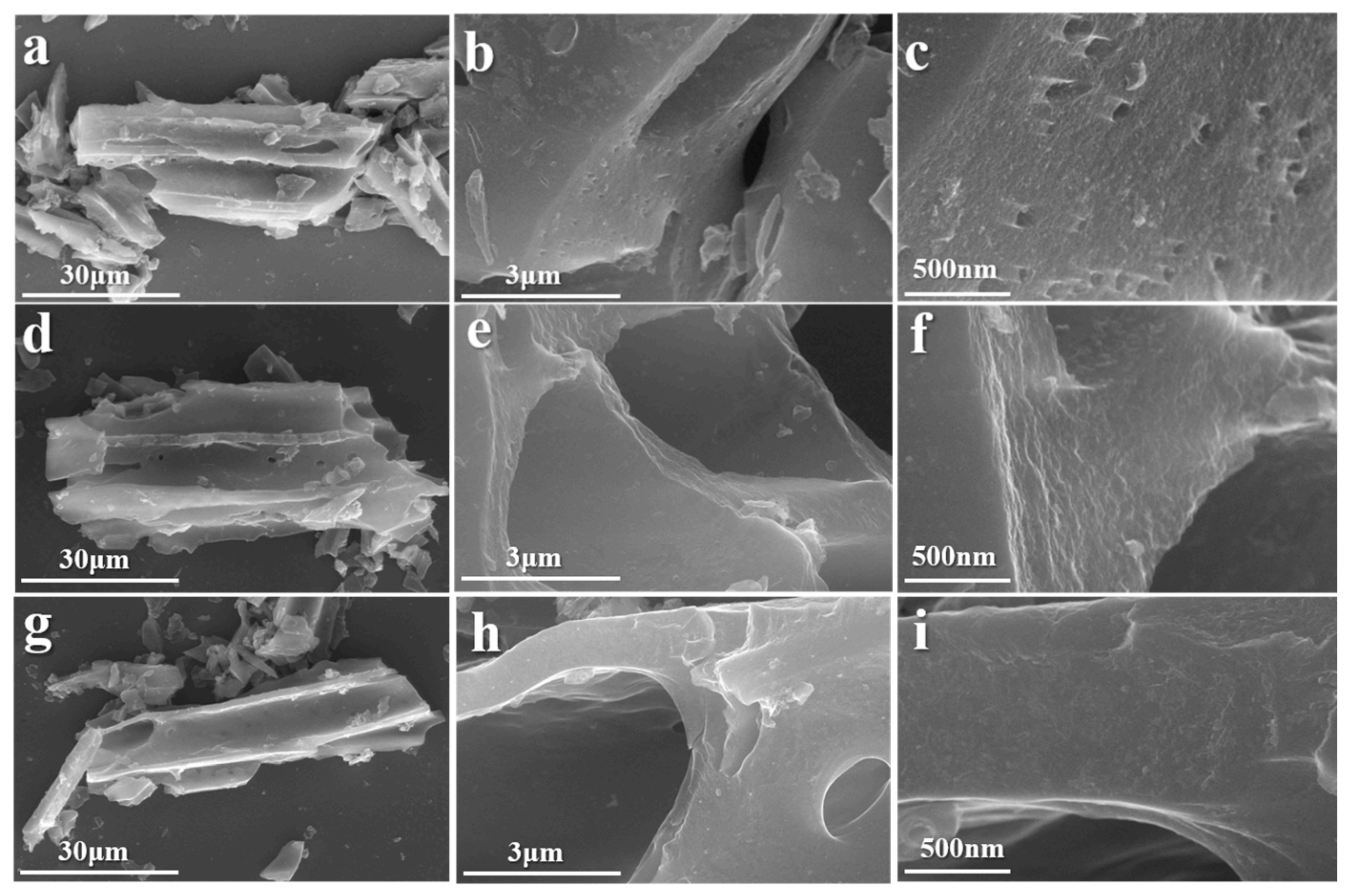
SEM images of (**a**–**c**) JPC-800-0, (**d**–**f**) JPC-900-0, and (**g**–**i**) JPC-1000-0.

The unactivated carbons prepared at 800, 900, and 1000 °C retained portions of the elongated/tubular morphology inherited from the *Suaeda salsa* precursor (Figure 1). JPC-800-0 displays more visible surface voids at high magnification, whereas the 900 and 1000 °C samples appear comparatively smoother and denser. These observations show that treatment temperature modifies the visible carbon morphology; however, no quantitative conclusion about temperature-dependent microporosity can be drawn from SEM alone [29,30].

**Figure 2:**
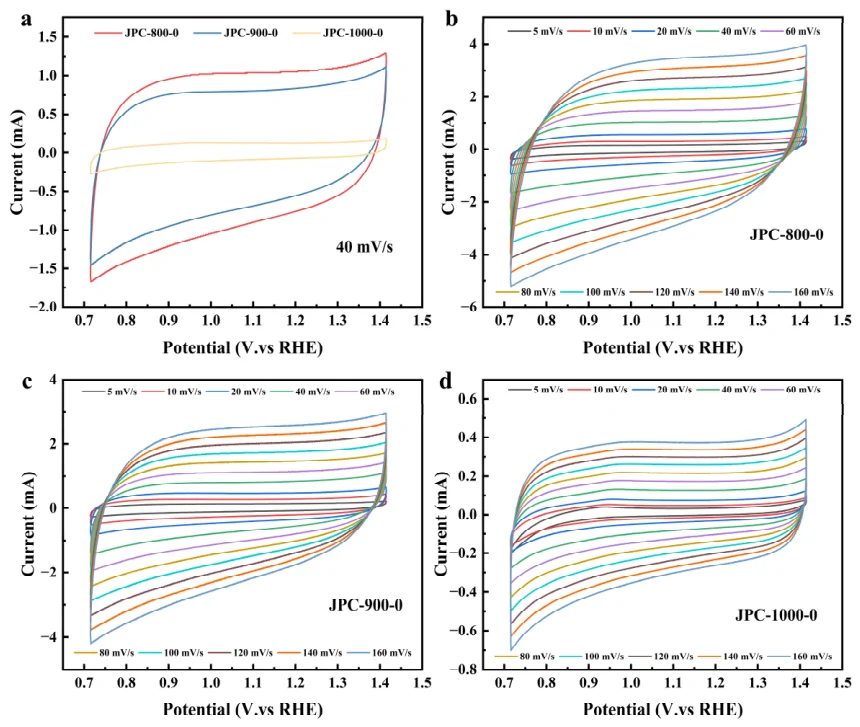
CV curves of *Suaeda salsa*-derived carbons prepared at 800, 900, and 1000 °C: (**a**) comparison at 40 mV s^−1^; (**b**–**d**) scan-rate-dependent CV curves of JPC-800-0, JPC-900-0, and JPC-1000-0. Potential values are shown vs. RHE after conversion from the Hg/HgO reference electrode, as described in Section 2.6.

CV measurements were carried out at scan rates of 5–160 mV s^−1^. Figure 2 compares the CV responses of JPC-800-0, JPC-900-0 and JPC-1000-0 collected at increasing scan rates. At a representative scan rate of 40 mV s^−1^ (Figure 2a), all three samples exhibit broad, approximately rectangular CV traces without well-defined redox peaks, which is characteristic of capacitive charge storage in porous carbon electrodes, although the noticeable slope and current increase toward the potential limits indicate non-ideal polarization/resistive contributions. Under the same conditions, JPC-800-0 gives the largest anodic and cathodic current densities and the largest enclosed CV area, followed by JPC-900-0, whereas JPC-1000-0 shows a markedly lower current response. The scan-rate-dependent CVs (Figure 2b–d) further show that the current magnitude increases systematically with scan rate for each sample while the overall quasi-rectangular shape is largely retained, indicating that the capacitive behavior is preserved at higher rates despite increasing polarization. Overall, based solely on the CV response, JPC-800-0 appears to possess the most favorable capacitive current among the three carbonization temperatures, suggesting that 800 °C is a more favorable pyrolysis temperature than 900 or 1000 °C.

**Figure 3:**
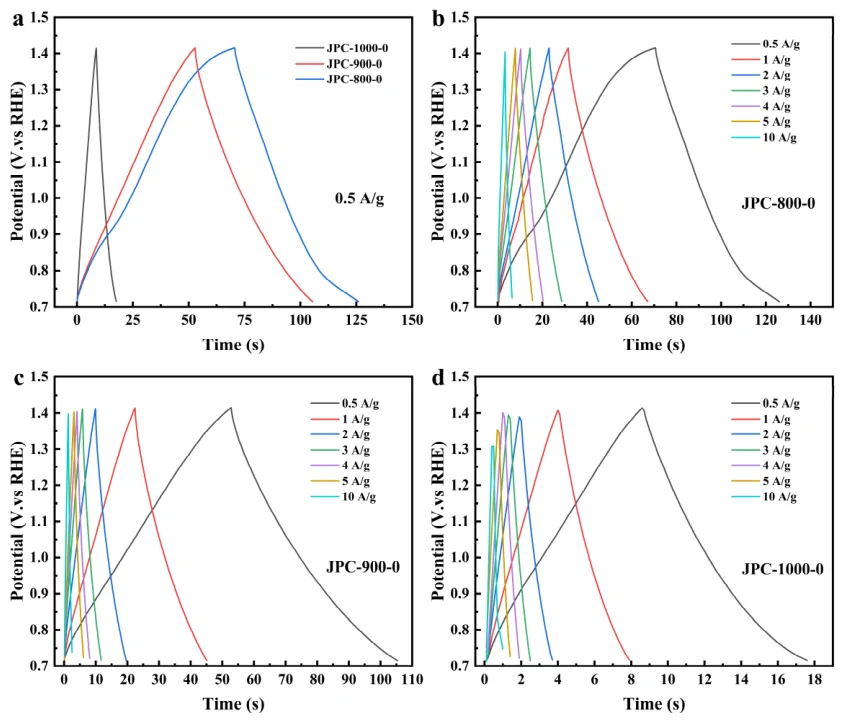
GCD curves of *Suaeda salsa*-derived carbons prepared at 800, 900, and 1000 °C: (**a**) comparison at 0.5 A g^−1^; (**b**–**d**) current-density-dependent GCD curves of JPC-800-0, JPC-900-0, and JPC-1000-0. Potential values are shown vs. RHE after conversion from the Hg/HgO reference electrode, as described in Section 2.6.

Figure 3a compares the GCD profiles of JPC-800-0, JPC-900-0, and JPC-1000-0 at the same current density of 0.5 A g^−1^ to evaluate the effect of pyrolysis temperature on their electrochemical performance. JPC-800-0 exhibits a substantially longer discharge duration than JPC-900-0 and JPC-1000-0, indicating a greater charge-storage capacity under the investigated conditions. Given the identical applied current density and comparable discharge potential windows, this extended discharge duration suggests a higher specific capacitance for JPC-800-0. The pronounced decrease in discharge duration at higher pyrolysis temperatures indicates that increasing the temperature from 800 to 900 or 1000 °C does not enhance the charge-storage performance of the resulting carbon materials. Therefore, 800 °C is identified as the optimal pyrolysis temperature among the three temperatures investigated, based on the GCD and CV. Consequently, for the subsequent preparation of *Suaeda salsa*-derived carbon materials, a pyrolysis temperature of 800 °C was consistently selected.

### 3.2. Effect of KOH Activation Ratio on the Pore Structure and Capacitive Performance

**Figure 4:**
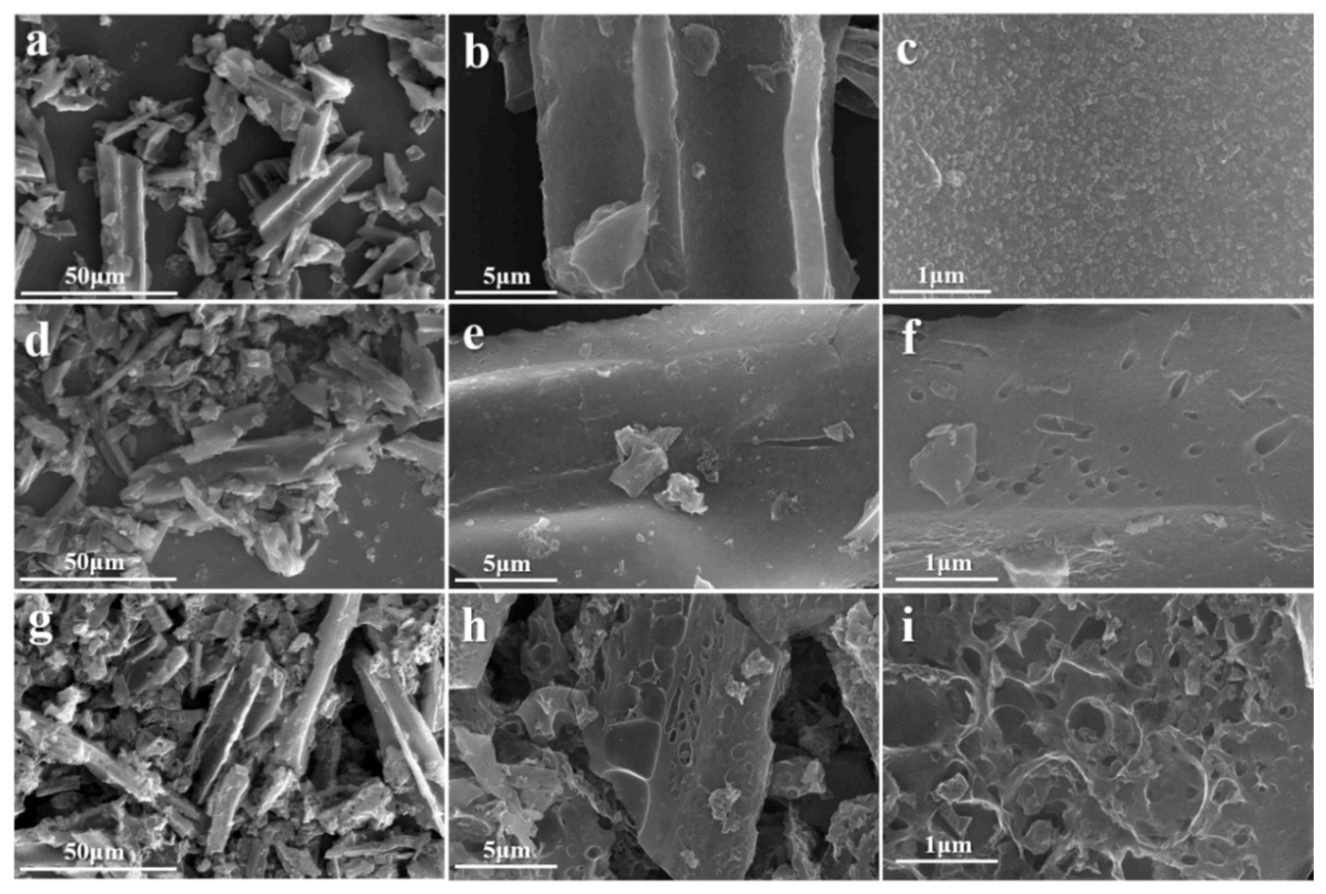
SEM images of (**a**–**c**) JPC-800-0.2, (**d**–**f**) JPC-800-0.5, and (**g**–**i**) JPC-800-1.

KOH activation was performed at KOH-to-pretreated-powder mass ratios of 0.2, 0.5, and 1.0 to prepare JPC-800-0.2, JPC-800-0.5, and JPC-800-1, respectively. Figure 4 shows that activation opens and etches the carbon framework. JPC-800-0.2 retains a comparatively compact surface, JPC-800-0.5 shows more distributed openings, and JPC-800-1 exhibits more pronounced surface disruption and larger visible voids. Nitrogen-sorption data for all four JPC-800-X samples are now available. They show that JPC-800-0.5 has the largest BET area and micropore volume, whereas JPC-800-1 has lower values than JPC-800-0.5 but higher values than JPC-800-0.2. Accordingly, excessive activation is discussed as a possible cause of less favorable pore-size distribution and framework damage, rather than as a pore-coalescence mechanism directly demonstrated by microscopy alone.

**Figure 5:**
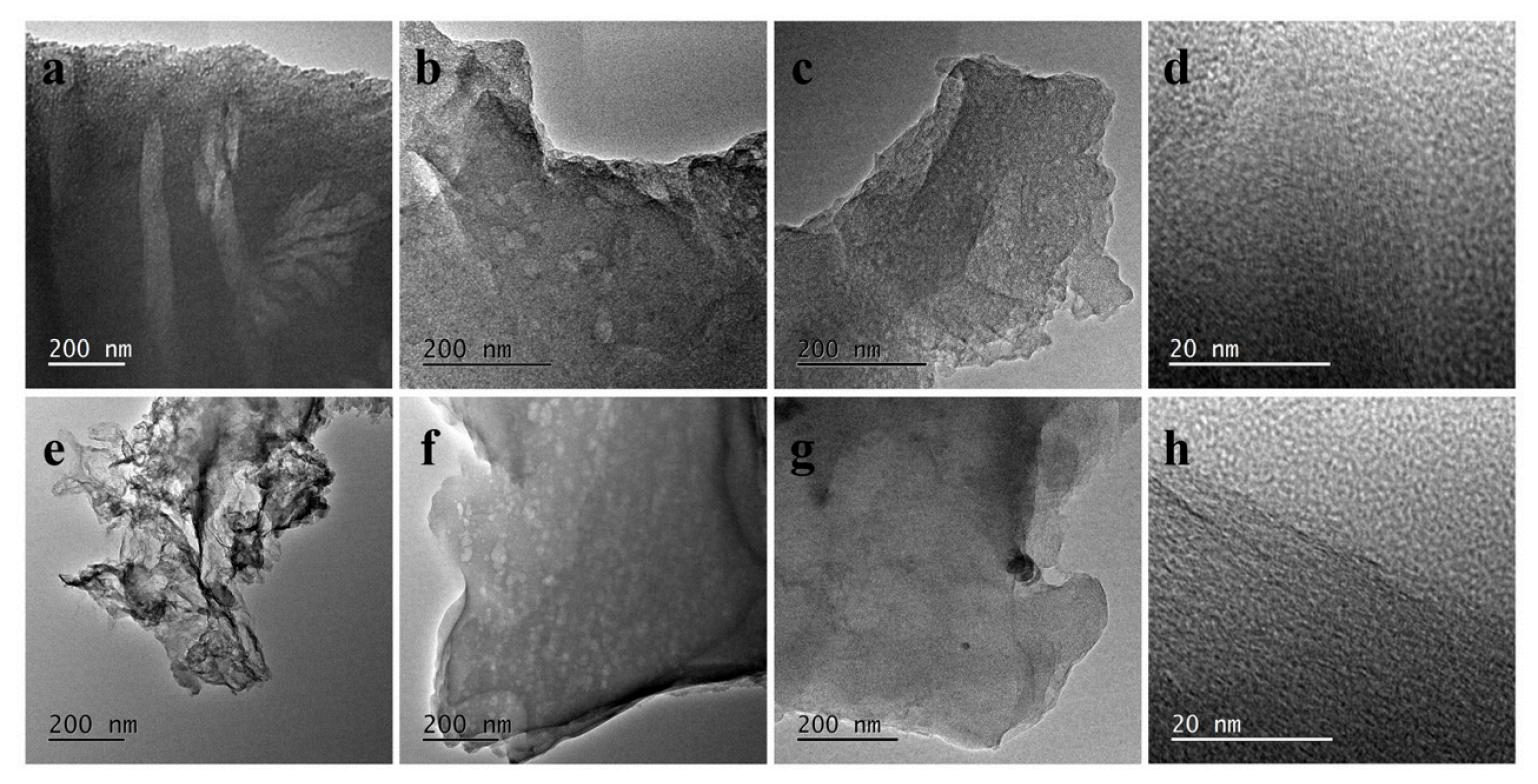
TEM images of (**a**–**c**) JPC-800-0 and (**e**–**g**) JPC-800-0.5; HRTEM images of (**d**) JPC-800-0 and (**h**) JPC-800-0.5.

As shown in Figure 5a–c, the unactivated JPC-800-0 sample consists of micrometer-scale carbon particles or stacked thick carbon domains with relatively dense surfaces and rough edges. The HRTEM image in Figure 5d shows short, curved lattice fringes, indicating limited local ordering within an otherwise disordered carbon framework.

After KOH activation, JPC-800-0.5 shows a more etched and wrinkled carbon framework (Figure 5e–g), while the HRTEM image in Figure 5h indicates disrupted short-range ordering in the observed region. These images support a morphological difference between the samples, but pore accessibility and micropore dimensions cannot be established from TEM contrast alone. The quantitative discussion of pore structure is therefore based primarily on the nitrogen-sorption data.

**Figure 6:**
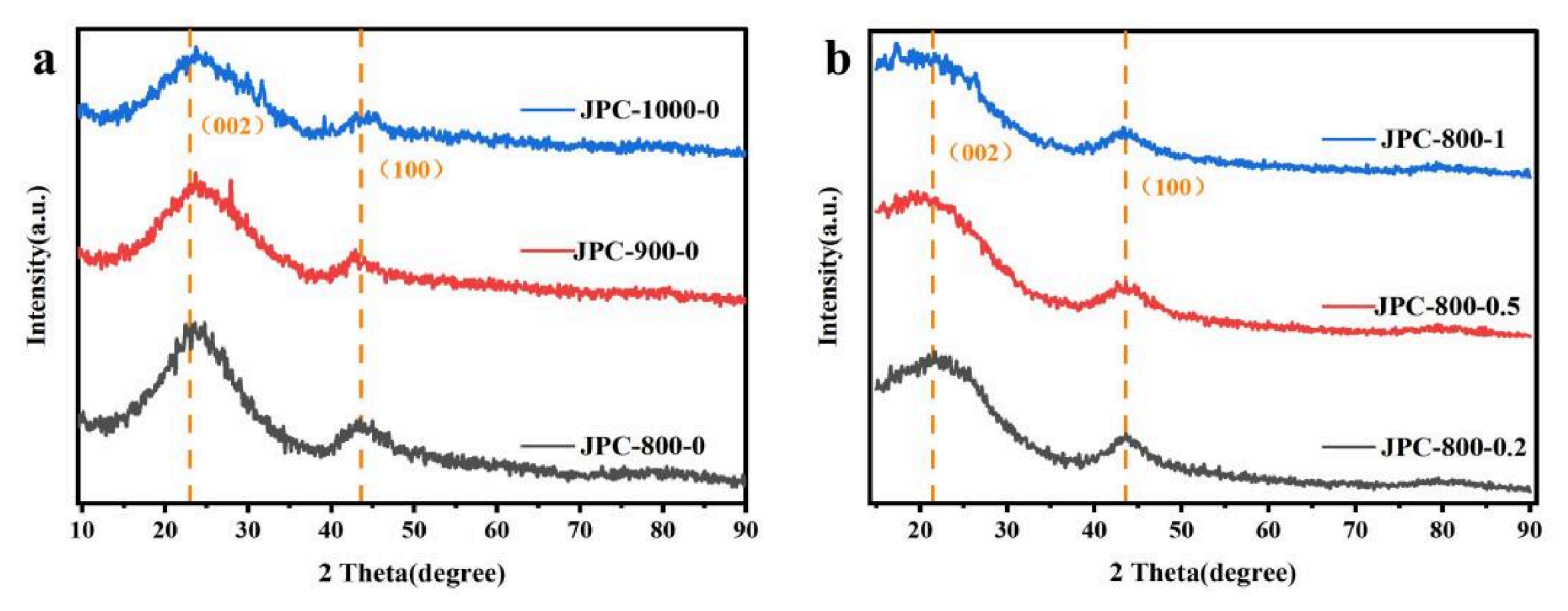
XRD patterns of *Suaeda salsa*-derived carbons prepared at (**a**) different pyrolysis temperatures and (**b**) different KOH-to-pretreated-powder mass ratios.

The XRD patterns of all JPC samples display a broad feature near 23°, commonly assigned to the (002) reflection of disordered/turbostratic carbon, together with a weak feature near 44° associated with the (100) reflection (Figure 6) [31,32]. The broad diffraction features indicate a low degree of long-range graphitic order. With increasing KOH ratio, the (002) feature shows a slight shift/broadening, suggesting modification of short-range carbon stacking during activation [33]. Because the diffraction bands are broad, the discussion is limited to qualitative structural changes rather than small quantitative differences in calculated interlayer spacing.

**Figure 7:**
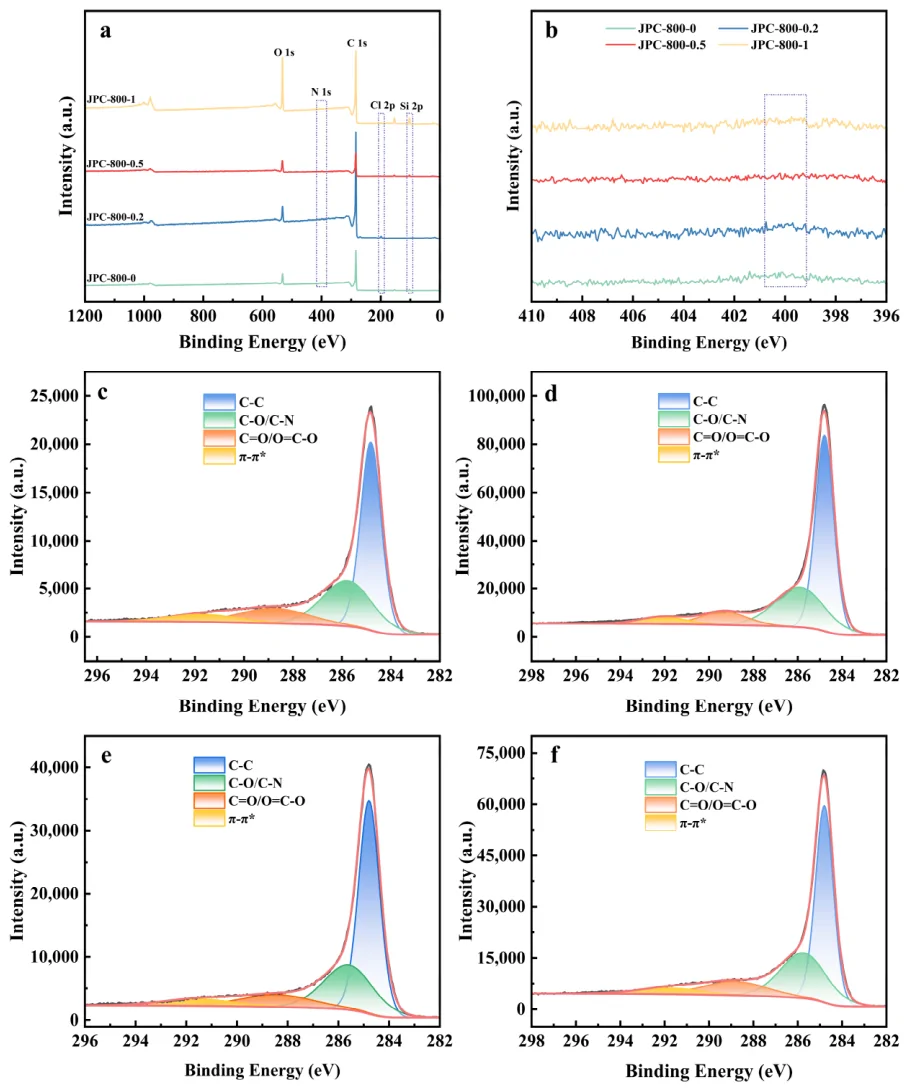
XPS spectra of JPC-800-X: (**a**) survey spectra; (**b**) high-resolution N 1s spectra; high-resolution C 1s spectrum of (**c**) JPC-800-0; (**d**) JPC-800-0.2; (**e**) JPC-800-0.5 and (**f**) JPC-800-1. (In (**b**), the purple box highlights the characteristic region of the N species. In (**c**–**f**), the black line represents the experimental (original) spectrum, while the red line shows the corresponding fitted curve).

XPS was used to compare the surface composition and chemical states of the JPC-800-X series (Figure 7 and Table 1). The survey spectra are dominated by C 1s and O 1s signals, with smaller Si 2p, N 1s, and Cl 2p contributions. JPC-800-0.5 shows an increased O 1s fraction (15.85 at.%) and the highest measured N 1s fraction (1.82 at.%), whereas JPC-800-1 has the lowest C fraction (73.20 at.%) and higher O and Si fractions. These changes are consistent with activation-dependent surface etching and changes in the amount of exposed oxygen-containing groups and inorganic residues. The C 1s components are assigned approximately to sp^2^ C–C (284.8 eV), overlapping C–O/C–N contributions (around 285.7 eV), C=O/O=C–O (around 288.6 eV), and the π–π* satellite (around 291.6 eV). Because the C–O/C–N envelope is overlapping and the N 1s signal is weak, the XPS data do not establish nitrogen doping as the main cause of the electrochemical differences.

The high-resolution N 1s signals (Figure 7b) are weak and close to the detection limit, so distinct nitrogen species could not be assigned reliably. No nitrogen-containing reagent was intentionally added during synthesis. The trace N signal may reflect residual biomass-derived nitrogen or surface contamination, but the available XPS data do not distinguish these possibilities. We therefore treat the N signal cautiously and do not attribute the electrochemical performance primarily to nitrogen incorporation.

**Figure 8:**
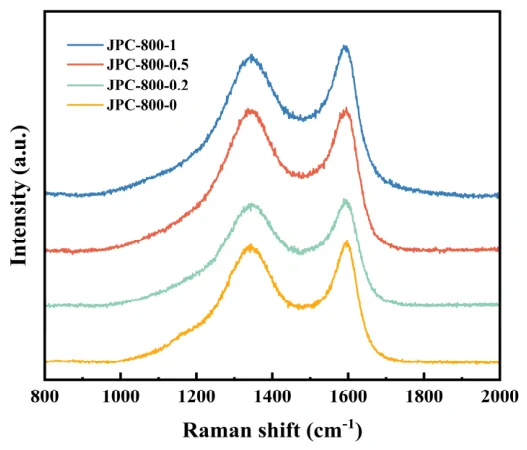
Raman spectra of JPC-800-X samples.

The Raman spectra show the characteristic D and G bands at approximately 1328 and 1598 cm^−1^, respectively (Figure 8). The reported I_G/I_D ratios are 0.98 for JPC-800-0, 1.00 for JPC-800-0.2, 1.02 for JPC-800-0.5, and 0.98 for JPC-800-1 (Table 2). These similar values indicate broadly comparable overall disorder across the activation series; Raman spectroscopy therefore supports the generally defect-rich, poorly graphitized character of the carbons but does not establish a substantial activation-induced increase in graphitic ordering.

**Figure 9:**
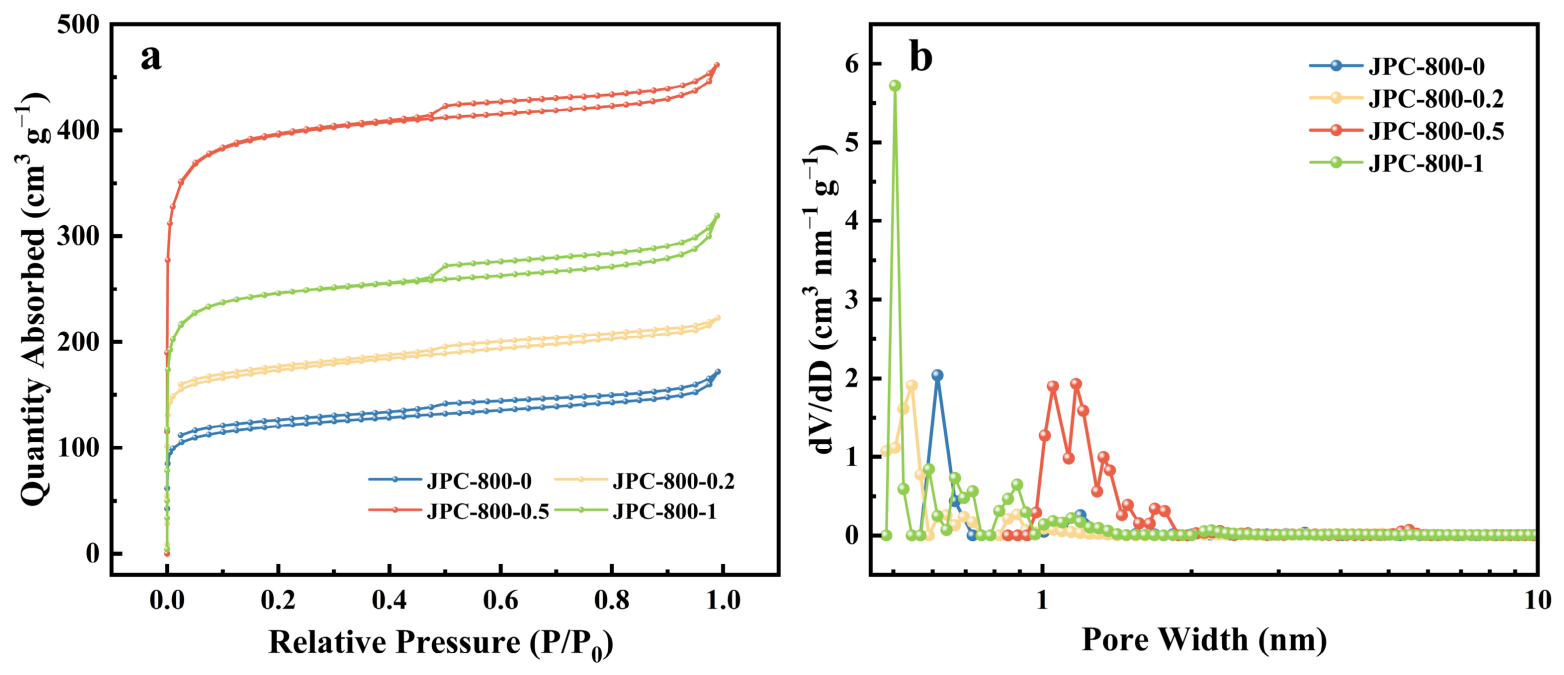
(**a**) N_2_ adsorption–desorption isotherms and (**b**) pore-size distributions of JPC-800-X.

Nitrogen adsorption–desorption analysis was used to compare JPC-800-0, JPC-800-0.2, JPC-800-0.5, and JPC-800-1 (Figure 9 and Table 3). The BET area increases from 457 m^2^ g^−1^ for JPC-800-0 to 837, 1536, and 1049 m^2^ g^−1^ for JPC-800-0.2, JPC-800-0.5, and JPC-800-1, respectively. JPC-800-0.5 also has the largest measured micropore volume (0.566 cm^3^ g^−1^) relative to 0.151, 0.226, and 0.306 cm^3^ g^−1^ for JPC-800-0, JPC-800-0.2, and JPC-800-1. The pore-size distributions suggest that JPC-800-0.5 combines a strong contribution in the approximately 0.9–1.5 nm region with a broader tail toward 2–5 nm, whereas JPC-800-1 shows a more concentrated sub-nanometer contribution and a smaller broader-pore population. These results are consistent with an intermediate activation ratio creating a more favorable balance between charge-storage micropores and electrolyte-transport pores. The lower capacitance of JPC-800-1 despite its larger BET area than JPC-800-0.2 further indicates that total surface area alone does not determine electrochemical performance.

**Figure 10:**
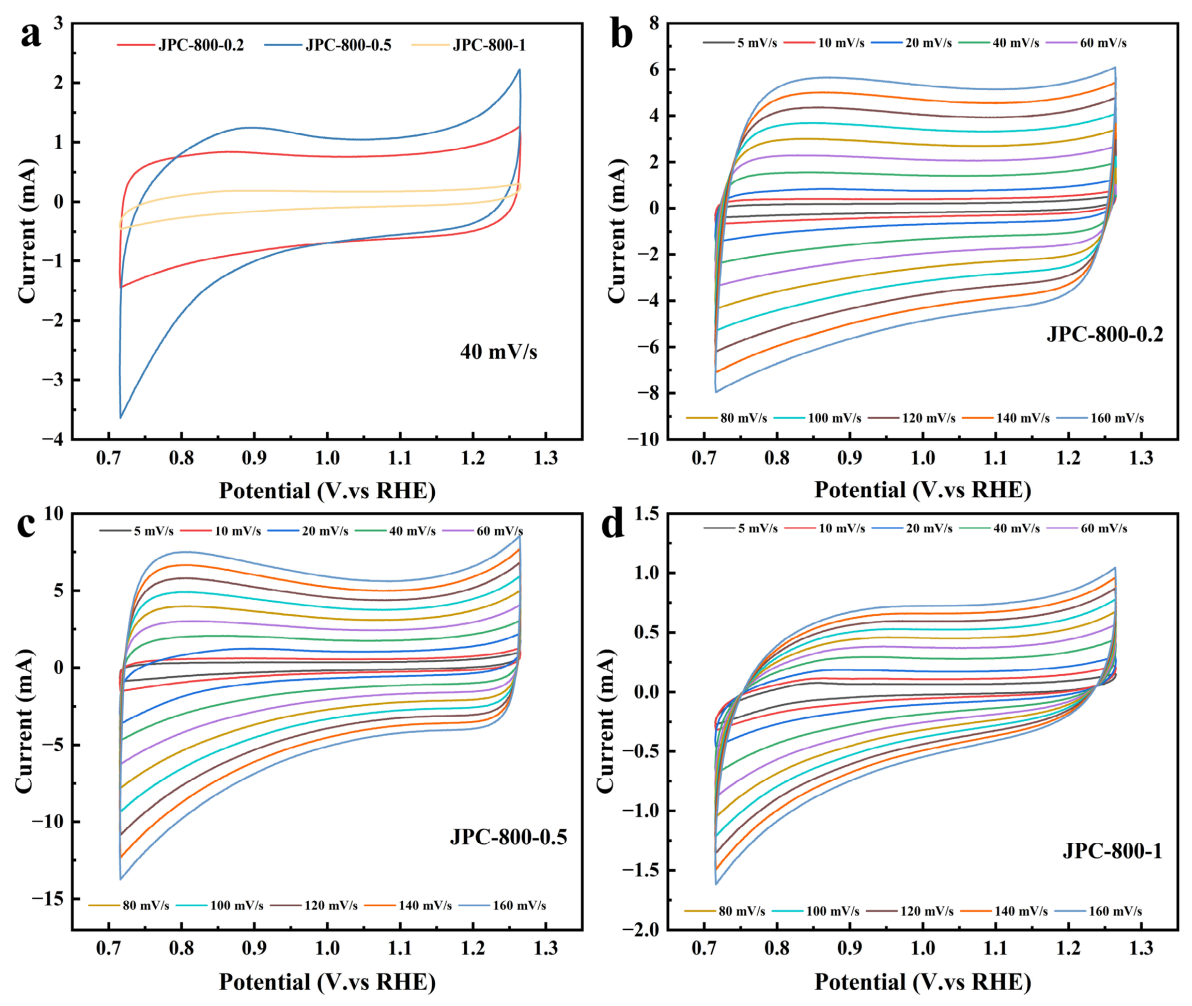
CV curves of *Suaeda salsa*-derived carbons prepared at different KOH-to-pretreated-powder ratios: (**a**) comparison at 20 mV s^−1^; (**b**–**d**) scan-rate-dependent CV curves of JPC-800-0.2, JPC-800-0.5, and JPC-800-1. Potential values are shown vs. RHE after conversion from the Hg/HgO reference electrode, as described in Section 2.6.

CV measurements were performed at scan rates of 5–160 mV s^−1^. At 20 mV s^−1^, the JPC-800-X electrodes show broadly quasi-rectangular responses characteristic of predominantly electric-double-layer charge storage (Figure 10a). JPC-800-0.2 and JPC-800-0.5 retain this profile more effectively as the scan rate increases, whereas JPC-800-1 shows greater distortion at high scan rates. Together with the pore-size data, the CV results suggest that the intermediate KOH ratio offers a more favorable balance between accessible surface area and ion-transport pathways among the tested conditions.

**Figure 11:**
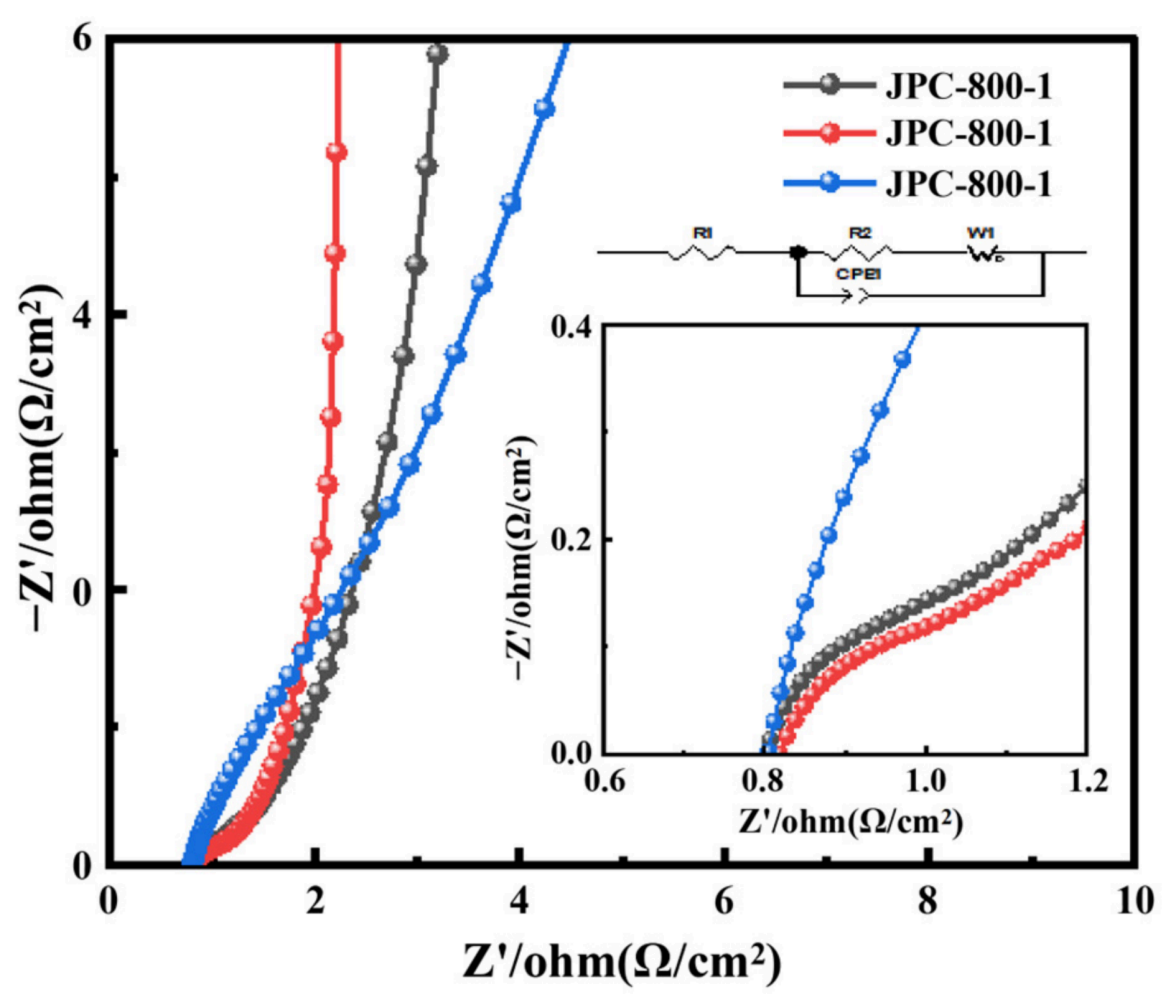
Nyquist plots of *Suaeda salsa*-derived carbons prepared at different KOH-to-pretreated-powder mass ratios (The inset shows a local magnification of the high-frequency region and the equivalent circuit diagram of the EIS).

EIS was used to compare the resistance and ion-transport behavior of the activated samples (Figure 11). The Nyquist plots show relatively small high-frequency arcs and increasingly steep low-frequency responses, with JPC-800-0.5 displaying the most nearly vertical low-frequency line among the three activated samples. This response is consistent with comparatively facile ion access and capacitive behavior. Notably, Raman analysis does not indicate a meaningful increase in graphitic ordering for JPC-800-0.5; therefore, the improved impedance response is more reasonably associated with the activation-induced increase in accessible porosity and pore connectivity than with enhanced graphitization. Accordingly, the EIS results are interpreted comparatively and qualitatively, without overinterpreting equivalent-circuit parameters.

**Figure 12:**
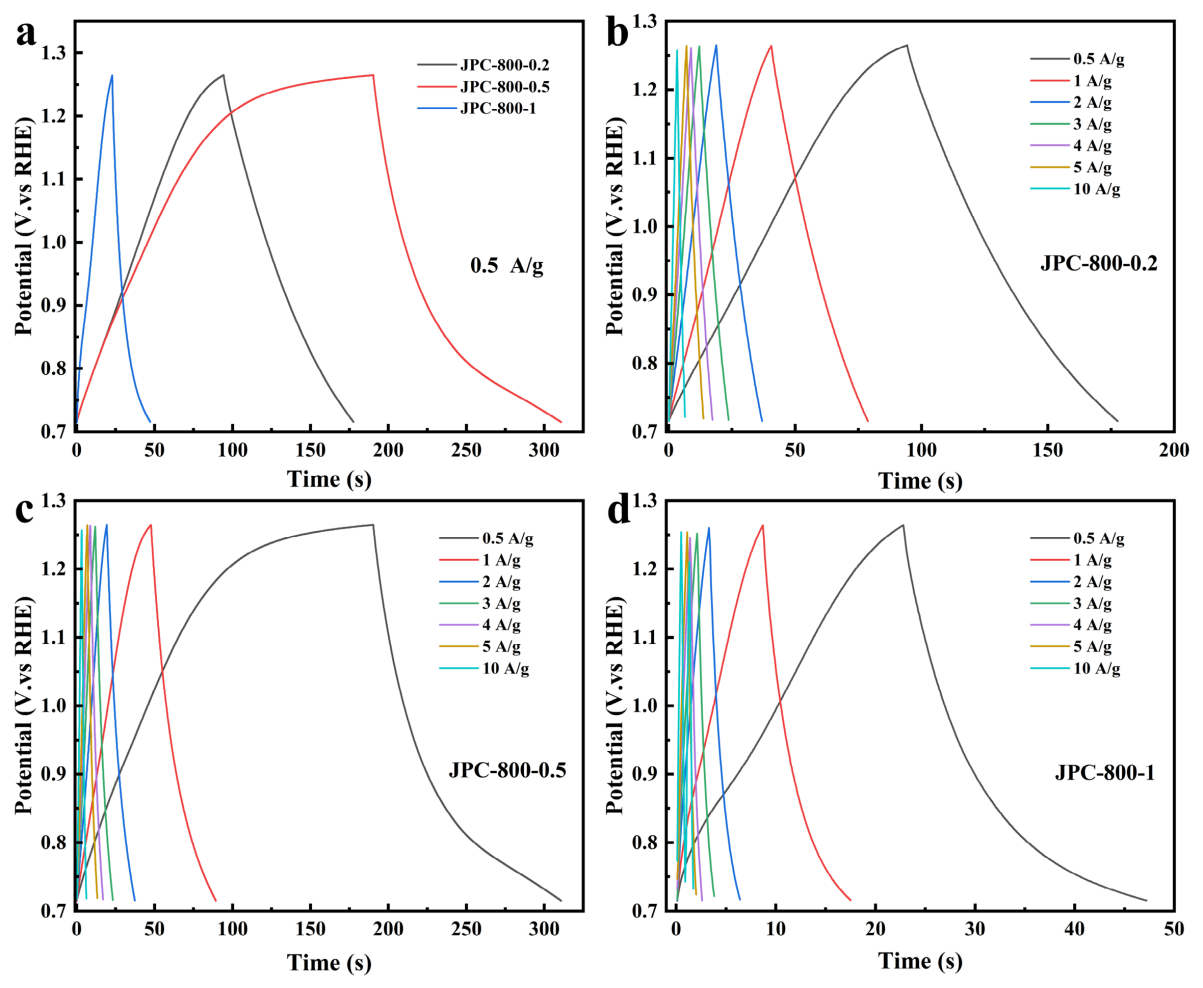
GCD curves of *Suaeda salsa*-derived carbons prepared at different KOH-to-pretreated-powder ratios: (**a**) comparison at 0.5 A g^−1^; (**b**–**d**) current-density-dependent GCD curves of JPC-800-0.2, JPC-800-0.5, and JPC-800-1. Potential values are shown vs. RHE after conversion from the Hg/HgO reference electrode, as described in Section 2.6.

GCD measurements were performed at 0.5–10 A g^−1^ in 6 mol L^−1^ KOH (Figure 12). At 0.5 A g^−1^, JPC-800-0.5 delivers 155 F g^−1^, compared with 86 F g^−1^ for JPC-800-0.2, 20 F g^−1^ for JPC-800-1, and approximately 44 F g^−1^ for unactivated JPC-800-0. The approximately 3.5-fold increase relative to JPC-800-0 is similar in magnitude to the increase in BET area and micropore volume. However, JPC-800-1 has a higher BET area and micropore volume than JPC-800-0.2 but a lower capacitance, showing that the accessible pore-size distribution and transport pathways are also important. The combined sorption and electrochemical data support an intermediate activation ratio as the best-performing condition among the conditions examined, without requiring pore coalescence to be treated as a directly observed mechanism.

**Figure 13:**
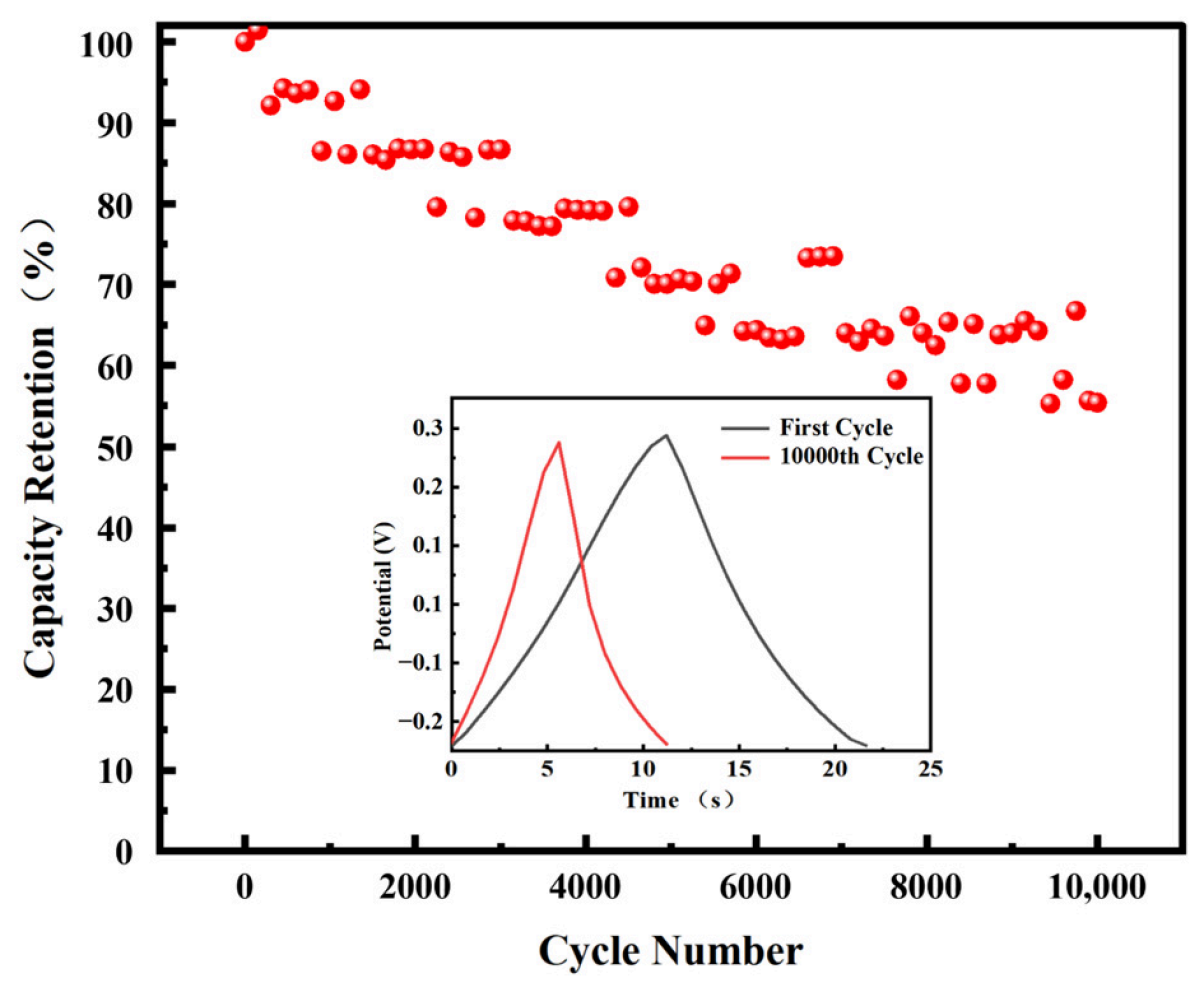
Cycling performance of JPC-800-0.5 at 5 A g^−1^, with representative GCD curves from the 1st and 10,000th cycles; the inset potential values are reported versus Hg/HgO (The red dots represent the capacity retention at different cycle numbers during the cycling test).

The cycling stability of JPC-800-0.5 was evaluated for 10,000 GCD cycles at 5 A g^−1^ (Figure 13). The specific capacitance decreases from approximately 95 F g^−1^ initially to 70 F g^−1^ after 5000 cycles (72% retention) and 54 F g^−1^ after 10,000 cycles (55% retention). These results demonstrate sustained capacitive operation but also reveal appreciable long-term fading. Because no post-cycling structural or chemical characterization was performed, a unique degradation mechanism cannot be assigned. Plausible electrode-level contributions include gradual loss of electrical contact within the carbon/conductive-additive/binder network, partial blockage or reduced accessibility of fine pores, and irreversible changes at oxygen-containing surface sites; these possibilities should be regarded as hypotheses rather than experimentally resolved mechanisms. The result further indicates that maximizing accessible pore volume and maintaining long-term electrode integrity are related but distinct optimization targets. The sample-comparison measurements and the cycling test discussed in this paragraph were performed in a three-electrode configuration. The reported gravimetric capacitances therefore describe the behavior of the active electrode material under laboratory testing conditions and should not be interpreted as the energy density or full-cell performance of a practical two-electrode supercapacitor. Long-term cycling durability remains the principal performance aspect requiring further improvement for this material system, particularly when compared with biomass-derived porous carbons reported with stronger long-cycle retention [34,35,36].

### 3.3. Evaluation of Electrochemical Properties of JPC-800-0.5 in Supercapacitors

**Figure 14:**
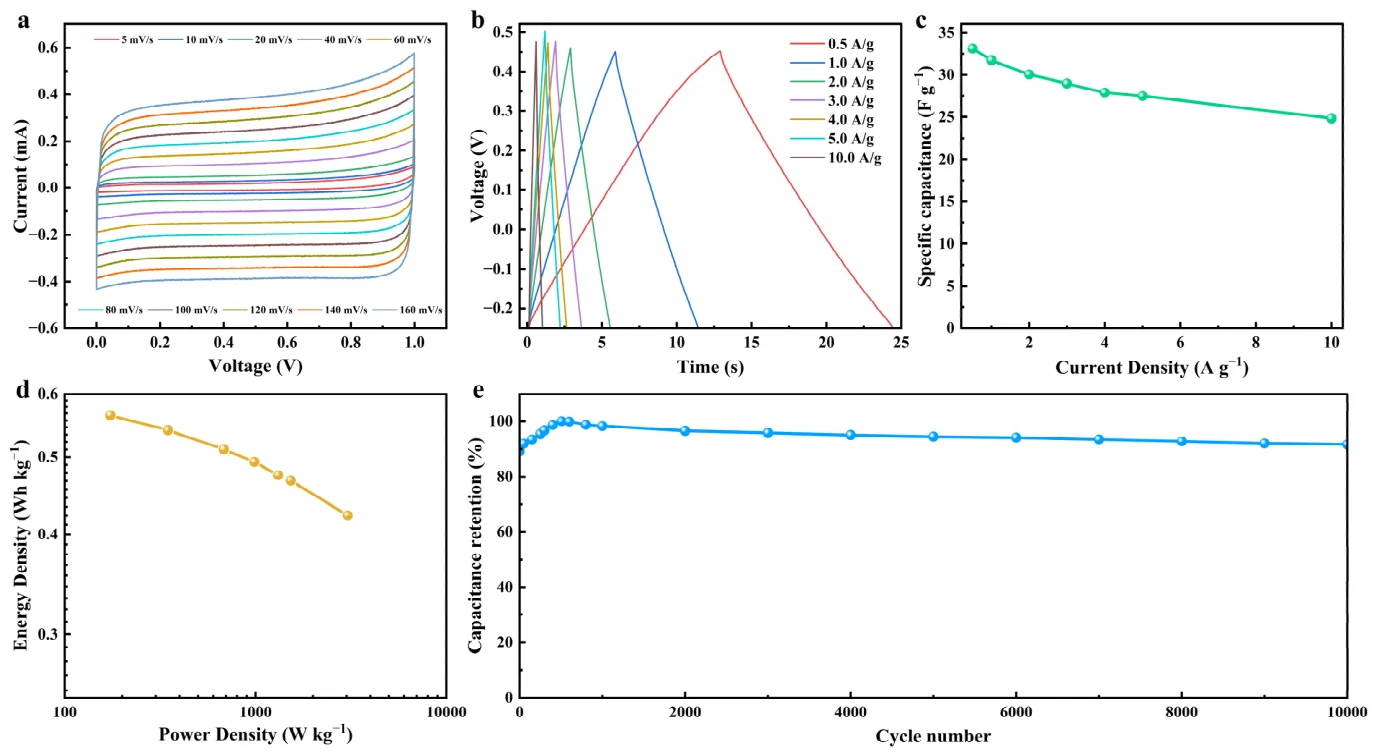
Electrochemical properties of JPC-800-0.5 in the KOH electrolyte: (**a**) CV curves; (**b**) GCD profiles; (**c**) specific capacitance; (**d**) Ragone plot; and (**e**) cycling stability at 5 A g^−1^.

To assess device-level behavior, JPC-800-0.5 was assembled in a symmetric two-electrode coin-cell configuration using 6 M KOH. Figure 14a shows the CV curves over the tested voltage window, while Figure 14b presents GCD profiles at different current densities. The reported device-specific capacitance is 33.13 F g^−1^ at 0.5 A g^−1^ and 24.84 F g^−1^ at 10 A g^−1^. The calculated energy density is 5.64 Wh kg^−1^ at a power density of 173.4 W kg^−1^. Table 4 compares these values with selected biomass-derived carbon devices. Because electrolyte composition, cell configuration, voltage window, and mass normalization differ across studies, the literature comparison is contextual rather than a strict ranking; the present device should not be described as outperforming most reported systems. The device cycling test shows 91.6% capacitance retention after 10,000 cycles at 5 A g^−1^. These device-level values are distinct from the three-electrode values and use the device-specific mass-normalization convention described in Section 2.6.

## 4. Conclusions

*Suaeda salsa*-derived porous carbons were prepared by thermal pretreatment, carbonization, and KOH activation. The combined microscopy, spectroscopy, nitrogen-sorption, and electrochemical data show that the activation ratio strongly affects pore development and charge-storage behavior. At a KOH-to-pretreated-powder ratio of 0.5, the BET area increases from 457 to 1536 m^2^ g^−1^, and the micropore volume increases from 0.151 to 0.566 cm^3^ g^−1^. The measured meso/macro-pore volume also increases modestly, from 0.083 to 0.116 cm^3^ g^−1^. These results are consistent with the development of additional microporous surface while retaining larger pores that can assist electrolyte transport.

Among the tested activation conditions, JPC-800-0.5 shows the highest three-electrode capacitance, reaching 155 F g^−1^ at 0.5 A g^−1^, which can be compared with approximately 44 F g^−1^ for JPC-800-0, 86 F g^−1^ for JPC-800-0.2, and 20 F g^−1^ for JPC-800-1. Although JPC-800-1 retains a relatively high BET area, its lower capacitance indicates that accessible pore architecture and framework preservation matter in addition to total surface area. In the three-electrode cycling test, JPC-800-0.5 retained approximately 72% of its initial capacitance after 5000 cycles and 55% after 10,000 cycles at 5 A g^−1^, showing that long-term durability remains an area for improvement.

In the symmetric two-electrode configuration, the reported device-specific capacitance was 33.13 F g^−1^ at 0.5 A g^−1^ and 24.84 F g^−1^ at 10 A g^−1^; the reported energy density was 5.64 Wh kg^−1^ at 173.4 W kg^−1^, with 91.6% retention after 10,000 cycles at 5 A g^−1^. These device-level values are distinct from the three-electrode results because the full-cell voltage window and total active-carbon mass define the device metrics.

Overall, moderate KOH activation produces the most favorable electrochemical response among the conditions tested by balancing micropore development, transport-accessible porosity, and preservation of the carbon framework. The results support a processing–pore structure–property relationship for *Suaeda salsa*-derived carbon, while the comparison with prior studies indicates that the device performance should be interpreted in the context of differences in electrolyte, cell design, and mass-normalization conventions.

## Figures and Tables

**Table 1:** Surface atomic concentrations of the detected elements determined by XPS (at.%; uncertainty ±0.1 at.%).

Sample	C (at.%)	O (at.%)	Si (at.%)	N (at.%)	Cl (at.%)
JPC-800-0	86.53	10.19	2.36	0.23	0.58
JPC-800-0.2	91.45	7.11	0	0.60	0.84
JPC-800-0.5	78.01	15.85	3.90	1.82	0.36
JPC-800-1	73.20	19.97	5.79	1.04	0

**Table 2:** Raman band positions and intensity ratios of the JPC-800-X samples.

Sample	D Band (cm^−1^)	G Band (cm^−1^)	I_G_/I_D_
JPC-800-0	1328	1598	0.98
JPC-800-0.2	1328	1598	1.00
JPC-800-0.5	1328	1598	1.02
JPC-800-1	1328	1598	0.98

**Table 3:** Porosity parameters of all JPC-800-X samples derived from N_2_ adsorption–desorption measurements.

Sample	BET Area (m^2^ g^−1^)	Micropore Volume (cm^3^ g^−1^)	Meso/Macro Volume (cm^3^ g^−1^)	Total Pore Volume (cm^3^ g^−1^)	Meso/Macro Fraction (%)
JPC-800-0	457	0.151	0.083	0.234	35.5
JPC-800-0.2	837	0.226	0.083	0.309	26.9
JPC-800-0.5	1536	0.566	0.116	0.682	17.0
JPC-800-1	1049	0.306	0.119	0.425	28.0

**Table 4:** Comparison with selected biomass-derived carbon supercapacitor devices.

Material	Device/Configuration	Reported Device-Level Performance	Cycling Stability	Ref.
JPC-800-0.5 (this work)	Symmetric two-electrode coin cell; 6 M KOH	33.13 F g^−1^ at 0.5 A g^−1^; 5.64 Wh kg^−1^ at 173.4 W kg^−1^	91.6% after 10,000 cycles at 5 A g^−1^	This work
Coffee-ground-derived hierarchical porous carbon	Symmetric two-electrode device; EMIMBF_4_ electrolyte	101 Wh kg^−1^ at 900 W kg^−1^	94% after 10,000 cycles at 10 A g^−1^	[34]
Phyllanthus emblica seed-derived activated carbon	Symmetric two-electrode cell; 1 M H_2_SO_4_	8.9 Wh kg^−1^ at 300 W kg^−1^	95% after 10,000 cycles	[35]
European deciduous-tree-derived activated carbon	Device-level supercapacitor test; see cited study for cell details	24 F g^−1^ at 0.25 A g^−1^	Stable cycling reported for 10,000 cycles	[36]

## Data Availability

The original contributions presented in this study are included in the article. Further inquiries can be directed to the corresponding author.

## Abbreviations

The following abbreviations are used in this manuscript:

EDLCs	Electric double-layer capacitors
BET	Brunauer–Emmett–Teller
SEM	Scanning Electron Microscopy
TEM	Transmission Electron Microscopy
XRD	X-ray Diffraction
XPS	X-ray Photoelectron Spectroscopy
CV	Cyclic Voltammetry
EIS	Electrochemical Impedance Spectroscopy
GCD	Galvanostatic Charge–Discharge
